# Characterization of the *Entamoeba histolytica* Ornithine Decarboxylase-Like Enzyme

**DOI:** 10.1371/journal.pntd.0000115

**Published:** 2008-01-02

**Authors:** Anupam Jhingran, Prasad K. Padmanabhan, Sushma Singh, Krishanpal Anamika, Abhijeet A. Bakre, Sudha Bhattacharya, Alok Bhattacharya, Narayanaswamy Srinivasan, Rentala Madhubala

**Affiliations:** 1 School of Life Sciences, Jawaharlal Nehru University, New Delhi, India; 2 Molecular Biophysics Unit, Indian Institute of Science, Bangalore, India; 3 School of Environmental Sciences, Jawaharlal Nehru University, New Delhi, India; Bose Institute, India

## Abstract

**Background:**

The polyamines putrescine, spermidine, and spermine are organic cations that are required for cell growth and differentiation. Ornithine decarboxylase (ODC), the first and rate-limiting enzyme in the polyamine biosynthetic pathway, is a highly regulated enzyme.

**Methodology and Results:**

To use this enzyme as a potential drug target, the gene encoding putative ornithine decarboxylase (ODC)-like sequence was cloned from *Entamoeba histolytica*, a protozoan parasite causing amoebiasis. DNA sequence analysis revealed an open reading frame (ORF) of ∼1,242 bp encoding a putative protein of 413 amino acids with a calculated molecular mass of 46 kDa and a predicted isoelectric point of 5.61. The *E. histolytica* putative ODC-like sequence has 33% sequence identity with human ODC and 36% identity with the *Datura stramonium* ODC. The ORF is a single-copy gene located on a 1.9-Mb chromosome. The recombinant putative ODC protein (48 kDa) from *E. histolytica* was heterologously expressed in *Escherichia coli*. Antiserum against recombinant putative ODC protein detected a band of anticipated size ∼46 kDa in *E. histolytica* whole-cell lysate. Difluoromethylornithine (DFMO), an enzyme-activated irreversible inhibitor of ODC, had no effect on the recombinant putative ODC from *E. histolytica.* Comparative modeling of the three-dimensional structure of *E. histolytica* putative ODC shows that the putative binding site for DFMO is disrupted by the substitution of three amino acids—aspartate-332, aspartate-361, and tyrosine-323—by histidine-296, phenylalanine-305, and asparagine-334, through which this inhibitor interacts with the protein. Amino acid changes in the pocket of the *E. histolytica* enzyme resulted in low substrate specificity for ornithine. It is possible that the enzyme has evolved a novel substrate specificity.

**Conclusion:**

To our knowledge this is the first report on the molecular characterization of putative ODC-like sequence from *E. histolytica*. Computer modeling revealed that three of the critical residues required for binding of DFMO to the ODC enzyme are substituted in *E. histolytica*, resulting in the likely loss of interactions between the enzyme and DFMO.

## Author Summary

*Entamoeba histolytica* is a unicellular protozoan parasite that infects about 50 million people each year and can cause potentially life-threatening diseases such as hemorrhagic colitis and extraintestinal abscesses. The infections are primarily treated by antiamoebic therapy. Drugs of choice for invasive amoebiasis are tissue-active agents, such as metronidazole, tinidazole, and chloroquine. Although drug resistance to *E. histolytica* does not appear to be a serious problem, there are occasional reports of failure with metronidazole, suggesting that clinical drug resistance may be developing. When identifying a drug target, it is important that the putative target be absent in the host, or, if it is present in the host, that the homologue in the parasite be substantially different from the host homologue so that it can be exploited as a drug target. Such is the case with the enzymes involved in polyamine biosynthesis, a pathway that has been exploited as a target to control disease caused by several parasites. We report, to our knowledge for the first time, molecular cloning, expression, and characterization of the ornithine decarboxylase from *E. histolytica,* a rate limiting enzyme in the polyamine biosynthesis pathway.

## Introduction

*Entamoeba histolytica* is a unicellular protozoan parasite that infects about 50 million people each year and may cause potentially life-threatening diseases such as hemorrhagic colitis and/or extraintestinal abscesses [1]. The infections are primarily treated by antiamoebic therapy. Drugs of choice for invasive amoebiasis are tissue-active agents such as metronidazole, tinidazole, and chloroquine [2]. Although drug resistance to *E. histolytica* does not appear to be a serious problem, there are occasional reports of failure with metronidazole suggesting the possibility of development of clinical drug resistance [3].

Polyamine biosynthetic pathway is the critical regulator of cell growth, differentiation, and cell death [4]–[6]. Polyamines are involved in nucleic acid packaging, DNA replication, apoptosis, transcription, and translation [7]. The polyamine biosynthetic pathway is a potential target for therapeutic agents against various hyperproliferative disorders, particularly cancer [8]–[10]. Given the importance of the polyamine biosynthetic pathway as a validated therapeutic target in protozoan parasites [11]–[14], we decided to further investigate this pathway in *E. histolytica* in the hope of extending our attempts at drug discovery to include this medically important parasite.

Ornithine decarboxylase (ODC; EC 4.1.1.17) is the first rate-limiting enzyme in polyamine biosynthesis, catalyzing the decarboxylation of L-ornithine to putrescine. This enzyme is found in a variety of systems ranging from bacteria [15] and protozoa [16] to plants [17] and mammals [18]. The rapid activation of the enzyme by various stimuli such as hormones, growth factors, or stress makes this enzyme a vital mediator in the regulation of polyamine pathway. ODC, like most amino acid decarboxylases, requires pyridoxal-5′-phosphate (PLP) as a cofactor [19]. *E. histolytica* ODC protein has been biochemically purified from trophozoites of the parasite [20]. Analytical electrophoresis revealed the presence of a major polypeptide of 45 kDa and scarcely noticeable amounts of two other proteins of 70 and 120 kDa. The major polypeptide exhibited amino-terminal sequence homology in the range of 40%–73% with ODCs of other organisms [20].

Biosynthesis of polyamines in parasites has been exploited as a target to control disease caused by several parasites with specific inhibitors of ODC such as α-difluoromethylornithine (DFMO), which is a structural analog of ornithine. DFMO has been found to be an efficient therapeutic agent against ODC up-regulation [13], [14], [21]–[23]. It is a specific and irreversible inhibitor of ODC, and previous studies have shown that DFMO inhibits growth of *Giardia lamblia* [24], *Acanthamoeba castellani* [25], *Plasmodium falciparum,* and some *Trypanosoma* species [13],[14],[21],[26] but has no inhibitory effect on *E. histolytica* ODC [20].

In this paper we report, to our knowledge for the first time, molecular cloning, expression, and characterization of a putative ODC-like sequence from *E. histolytica,* the parasitic protozoan responsible for amoebiasis. ODC is a PLP-dependent enzyme, and in the present work the ability of *E. histolytica* putative ODC to form complexes with PLP and DFMO was investigated using modeling of the three-dimensional structure.

## Materials and Methods

### Reagents

Restriction enzymes Pfu and Taq DNA polymerases were obtained from MBI Fermantas. All other chemicals were of analytical grade and were available commercially.

### Parasite and culture conditions

All experiments were carried out with *E. histolytica* strain HM-1:IMSS clone 6, which was obtained from William A. Petri (University of Virginia). The cells were maintained and grown in TYI-33 medium supplemented with 15% adult bovine serum, 2% Diamond's vitamin mix, and antibiotic (0.3 units/ml penicillin and 0.25 mg/ml streptomycin). Cell viability was determined by microscopy using a trypan blue dye exclusion test. Experiments were conducted with cells that showed >90% viability.

### Cloning of putative *ODC*-like gene sequence from *E. histolytica*

A ∼1,242 base pair fragment was amplified from the genomic DNA of *E. histolytica* using a sense primer with flanking BamH I site (underlined), 5′-CGCGGATCC ATGAAACAAACATCTCTAGAAG-3′, which codes for amino acid sequence MKQTSLE at position 1–21 and one extra base, G, and the antisense primer with a flanking Xho I site (underlined), 5′- CCGCTCGAGAGCATAGTGTGGAATACCAT-3′, which codes for amino acids GIPHYA at position 1,220–1,239 with two extra bases, A and T. Polymerase chain reaction was performed in a 50 µl reaction volume containing 150 ng of genomic DNA, 25 pmol each of gene-specific forward and reverse primers, 200 µM of each dNTPs, 2.5 mM MgCl_2_, and 2.5 units of Taq DNA Polymerase (MBI Fermentas). PCR cycling conditions were as follows; 94°C for 10 min, followed by 35 cycles of 94°C for 1 min, 47°C for 45 sec, 72°C for 1:30 min. A final extension was carried out for 10 min at 72°C. A single 1,242 bp PCR product was obtained and subcloned into pTZ57R T/A vector (Promega, Madison, USA) and subjected to automated sequencing. Sequence analysis was performed by DNAstar, whereas comparisons with other sequences of the database were performed using the search algorithm BLAST [27]. Multiple alignments of amino acid sequences were performed using CLUSTAL W (http://www.ebi.ac.uk/clustalw/). The phylogenetic tree was constructed using PHYLIP style treefile produced by CLUSTAL W. The ∼1242-bp DNA fragment, amplified by Pfu polymerase (MBI Fermentas), was also cloned into the BamH I-Xho I site of pET 30a vector (Novagen). The recombinant construct was transformed into BL21 (DE3) strain of *E. coli.*

### Expression and purification procedure

Expression from the construct pET30a-ODC-like sequence was induced at O.D. of 0.3 with 1 mM IPTG (isopropyl β-D-thiogalactoside) (Sigma) at 37°C for different time periods. Bacteria were then harvested by centrifugation and the cell pellet was resuspended in binding buffer (50 mM sodium phosphate buffer, pH 7.5; 10 mM imidazole, pH 7.0; 300 mM sodium chloride; 2 mM phenylmethylsulphonyl fluoride (PMSF); and 30 µl protease inhibitor cocktail). Lysozyme (100 µg/ml) was added to the cell suspension and kept on a rocking platform for 30 min at 4°C. The resulting suspension was sonicated six times for 20 s with 1 min intervals. The lysate was centrifuged at 20,000*g* for 30 min at 4°C. The resulting supernatant, which contained protein, was loaded onto a pre-equilibrated Ni-NTA agarose beads (Ni^2+^-nitrilotriacetate)-agarose beads (Qiagen). The mixture was kept on a rocking platform for 2 h at 4°C. It was centrifuged at 400 g for 30 min at 4°C. The supernatant was discarded and pellet was washed thrice with wash buffer (50 mM sodium phosphate buffer, pH 7.5; 50 mM imidazole, pH 7.0; 300 mM sodium chloride; 2 mM phenylmethylsulphonyl fluoride [PMSF]; and 30 µl protease inhibitor cocktail). The protein was eluted with increasing concentrations of imidazole, pH 7.0. The imidazole was removed by dialysis in 20 mM sodium phosphate buffer, pH 7.5. The purified protein was aliquoted and stored at −80°C.

### Nucleic acid isolation, pulsed-field gradient gel electrophoresis, and hybridization analysis

Genomic DNA was digested with the enzymes XhoI and HindIII and subjected to electrophoresis in 0.8 % agarose gels. The fragments were transferred to nylon membranes (Amersham Pharmacia Biotech) and subjected to Southern blot analysis. For Northern blot analysis, 15 µg of total RNA was fractionated by denaturing agarose gel electrophoresis and transferred onto nylon membrane following standard procedures. Pulsed-field gradient gel electrophoresis (PFGE) was carried out essentially as described earlier [28]. The agarose blocks containing the cells were subjected to PFGE in 1.2% agarose gels using the Gene Navigator system (Pharmacia). The pulse conditions used were 70 s for 15 h, 120 s for 14 h, and 200 s for 7 h at 5.5 V cm^−1^. *Saccharomyces cerevisiae* chromosomes were used as size markers. Following the transfer of DNA, RNA, and chromosomes onto nylon membranes, the nucleic acids were UV cross-linked to the membrane in a Stratagene UV cross-linker. Prehybridization was done at 65°C for 4 h in a buffer containing 0.5 M sodium phosphate; 7% SDS; 1mM EDTA, pH 8.0; and 100 µg/ml sheared denatured salmon sperm DNA. The blots were hybridized with denatured α-[PPPP^32^P]-dCTP-labeled DNA probe (PCR probe described for the *E. histolytica* putative ODC*-*coding region) at 10PP^6P^ cpm/ml, which was labeled by random priming (NEB BlotPPKit, New England Biolabs). Membranes were washed, air-dried, and exposed to an imaging plate. The image was developed by PhosphorImager (Fuji film FLA-5000, Japan) using Image Quant software (Amersham Biosciences).

### Preparation of crude extract of *E. histolytica* for ODC assay

*E. histolytica* (1×10^6 ^cells) were harvested by centrifugation at 16,000 *g* at 4°C for 10 min, washed with phosphate-buffered saline, pH 7.4. The cell pellet was resuspended in lysis buffer (100 mM Tris-Cl, pH 7.5; 150 mM sodium chloride; 2 mM PMSF; 2 mM iodoacetamide; 2 mM EDTA; 2.5 mM parahydroxymercuricbenzoic acid; 2 mM ethylene glycol-bis (amino ether); and 10 µg/ml proteinase cocktail) and incubated on ice for 10 min. The cells were lysed by freeze-thaw in liquid nitrogen and subjected to sonication for 10 sec with 1 min interval at 4°C, thrice. The lysate was centrifuged at 15,000*g* for 30 min at 4°C and the supernatant was used for ODC assay, polyamine estimation, and Western blot analysis as mentioned below.

### ODC assay

ODC activity was assayed by following the release of ^14^CO_2_ from L- [-^14^C] ornithine [29]. The standard assay mixture containing the supernatant, 200 µM PLP; 12.5 mM DTT; 250 mM Tris, pH 7.5; 2 mM ornithine; and 3 µCi of the radiolabeled ornithine were incubated at 37°C for 1 h. The reaction was terminated by injecting 5 N H_2_SO_4. _Activity is expressed in enzyme units in which one unit is nmol of CO_2 _/mg protein/h. The assay was repeated thrice. Protein concentrations were determined by the method of Bradford [30] using bovine serum albumin as standard.

### Polyamine analysis

Quantitative determination of polyamines in crude lysates of *E. histolytica* was performed by C18 reversed-phase high performance liquid chromatography after precolumn derivatization with dansyl chloride [31]. The results were based on three separate determinations.

### Antibody production

The purified recombinant putative ODC-like protein (20 µg) was subcutaneously injected in mice using Freund's complete adjuvant, followed by two booster doses of recombinant putative ODC-like protein (15 µg) with incomplete adjuvant at 2 wk intervals to produce polyclonal antibody against the recombinant putative ODC-like protein. The mice were bled after 2 wk after the second booster, and sera were collected and used for Western blot analysis.

### Western blot analysis

Recombinant putative ODC-like protein and cell lysate (100 µg of protein) from *E. histolytica* were fractionated by SDS/PAGE blotted on to nitrocellulose membrane using electrophoretic transfer cell (Bio-Rad). Western blot analysis was carried out using the ECL (enhanced chemiluminescence) kit (Amersham Biosciences) according to the manufacturer's protocol. Anti-polyhistidine (mouse IgG2a isotype, Sigma) and polyclonal antibody (1∶500 dilution) against purified recombinant *E. histolytica* putative ODC generated in mice were used for Western blot analysis.

### Structural modeling of *E. histolytica* putative ODC and analysis of the binding site

**Figure 1:**
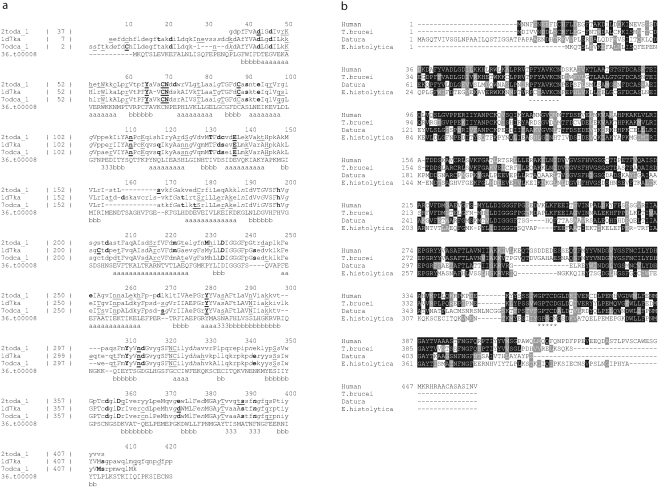
Structure-based alignment of ODCs from *T. brucei* (2toda) (ExPASy accession number: P07805), *H. sapiens* (1d7ka) (ExPASy accession number: P11926), *M. musculus* (7odca) (ExPASy accession number: P00860), and *E. histolytica* (36.t00008) (GenBank accession number: Q58P26). (A) The structural environments of the residues in the known structures are encoded in the representation: Upper case letters are solvent-inaccessible amino acids; lower case letters are solvent accessible; italicized letters are residues with a positive φ (one of the Ramachandran angles); bold letters indicate a side chain hydrogen bonded to the main chain amide; underlined letters indicate a side chain hydrogen bonded to the main chain carbonyl. The numbers within parenthesis represent the first residue of the given protein in a block. Conserved α-helical, 3_10_ helical, and β-strand regions are indicated at the end of every alignment block as a, 3, and b respectively. Figure produced using JOY [34]. (B) Multiple sequence alignment of putative ODC-like sequence from *E. histolytica* (GenBank accession number AAX35675), *T. brucei* ODC (GenBank accession number AAA30219), human ODC (GenBank accession number AAA59967), and *Datura stramonium* ODC (GenBank accession number CAA61121) using ClustalW. The amino acids are numbered to the left of the respective sequences. Residues that are identical or similar to other ODCs are indicated in black showing complete identity, and grey when they are conserved in at least three sequences. Short dashed line below the sequence represents the pyridoxal phosphate binding site. Line of asterisks (*) represents the DFMO binding site.

The structure of the *Trypanosoma brucei* ODC mutant in complex with DFMO [32] was used as a template to model *E. histolytica* ODC. In this mutant structure, lysine 69 has been mutated by alanine (K69A). STAMP (structural alignment of multiple proteins) [33], was used for structural alignment of three ODCs from *T. brucei* (2TOD) (ExPASy [http://expasy.org/] accession number: P07805), *H. sapiens* (1D7K) (ExPASy accession number: P11926) and *M. musculus* (7ODC) (ExPASy accession number: P00860). Later, the program JOY (version 5) [34] was used to align and merge three structurally aligned ODCs with the sequence of *E. histolytica* putative ODC such that properties of both the structure-based alignment for the homologues of known three-dimensional structures and the sequence-based alignment involving *E. histolytica* putative ODC are reflected in the final alignment used for modeling (Figure 1A). JOY represents structural information and annotates each amino acid residue according to its structural environment. JOY uses local structural features calculated from the atomic coordinates in a PDB file. The three-dimensional model of *E. histolytica* putative ODC in complex with PLP and DFMO has been built based on the crystal structure of *T. brucei* ODC by using the program MODELLER [35]. MODELLER generates a three-dimensional structure of a given protein sequence (target) based primarily on its alignment to one or more proteins of known structure (template/templates). The modeling process consists of fold assignment, target-template alignment, structure building, and evaluation. MODELLER implements comparative protein structure modeling by satisfying spatial restraints [36],[37] and performs tasks such as de novo modeling of loops, comparison of protein structures, optimization of various models of protein structures, etc. Interactive graphics like SYBYL (Tripos, St. Louis, Missouri, United States) was used for energy minimization of the modeled structure to relieve the short contacts, if any.

### Energy minimization

Modeled *E. histolytica* putative ODC was subjected to energy minimization using the AMBER force field [38] encoded in the SYBYL software. Energy minimization was done in order to rectify all stereochemical inconsistencies and short contacts that may be present in the initial model.

## Results

### Sequence analysis and genomic organization

In order to clone the gene encoding putative ODC-like gene, PCR was performed using specific oligonucleotides (as described in the Methods section), whose sequence was based on Genome Sequencing Project of *E. histolytica* (http://www.tigr.org). Examination of the *E. histolytica* database predicts a single ODC gene (http://pathema.tigr.org). A single open reading frame consisting of ∼1,242 bp was obtained, cloned, and sequenced. (*E. histolytica ODC* gene, GenBank [http://www.ncbi.nlm.nih.gov/Genbank/] accession number AY929249).

The open reading frame coded for a putative polypeptide of 413 amino acids, with a predicted molecular mass of ∼46 kDa. The predicted isoelectric point (pI) of *E. histolytica* putative ODC-like protein (GenBank accession number AAX35675) was determined to be 5.61, comparable to those of proteins from *L. donovani* (GenBank accession number P27116) (pI 5.29), and *T. brucei* (GenBank accession number AAA30219) (pI 5.46) (Figure 1). There was only 33% sequence identity with human ODC (GenBank accession number AAA59967), 32% identity with *T. brucei* (GenBank accession number AAA30219), and 36% identity with *Datura stramonium* ODC (GenBank accession number CAA61121) sequences (Figure 1B). The sequences of the mammalian ODC has some highly conserved amino acids and regions that are reported to be essential for catalytic activity and dimerization [39]–[41], and these were also found in the putative ODC-like sequence of *E. histolytica*. The putative ODC-like sequence was 413 amino acids smaller than ODCs from *T. brucei*, *Homo sapiens*, and *D. stramonium* (Figure 1B). The residue that is essential for dimerization of ODC monomers, mediated by glycine-387 in mammals [42], was found to have equivalents in the sequence of the *E. histolytica* putative ODC-like protein at position glycine-361. The sequence motif PFYAVKCN at position 64–71 of mammalian ODC, which contains the lysine-69 residue to which the cofactor pyridoxal-5′-phosphate binds, is present at position 53–60 of the *E. histolytica* putative ODC-like protein, although with changes of phenylalanine to cysteine and tyrosine to phenylalanine. The region GPSCNGSD at position 331–338 in the *E. histolytica* putative ODC-like protein is probably equivalent to consensus sequence GPTCDGLD of the ODC sequences of various eukaryotes. This sequence contains cysteine-360 in mammalian ODC, which is the major binding site of α-difluoromethylornithine (DFMO) [40]. The corresponding cysteine-334 is conserved in *E. histolytica* putative ODC-like protein. The overall amino acid homology of *E. histolytica* putative ODC with the mammalian ODC is low, but highly conserved signature motifs responsible for dimerization and catalytic activity were present. Another signature sequence, as predicted by PROSITE, D(I/V)GGGF, is present across varied sequences without exception. Other highly conserved amino acid stretches, i.e., FDCAS, EPGR, FNGF, and GAYT, are also consistently conserved, though the functional significance of these stretches is not known.

**Figure 2:**
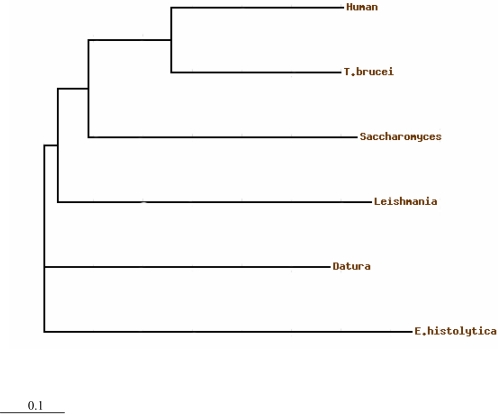
Phylogenetic tree using the amino acid sequences of putative ODC from *E. histolytica* and ODCs from other organisms. The TreeView program in the ClustalW displayed the phyletic trees derived from the multiple alignments.

A phylogenetic tree was constructed using the *E. histolytica* putative ODC-like sequence and other representative ODC sequences (Figure 2). The nearest homologue to the amoebic protein, as revealed by the tree, is the plant *D. stramonium*. The human ODC sequence seems to be farthest from the amoebic one. Among kinetoplastids, *L. donovani* appears to be the closest homologue, while *Trypanosoma* not clustering with *L. donovani*, is quite distantly related.

**Figure 3:**
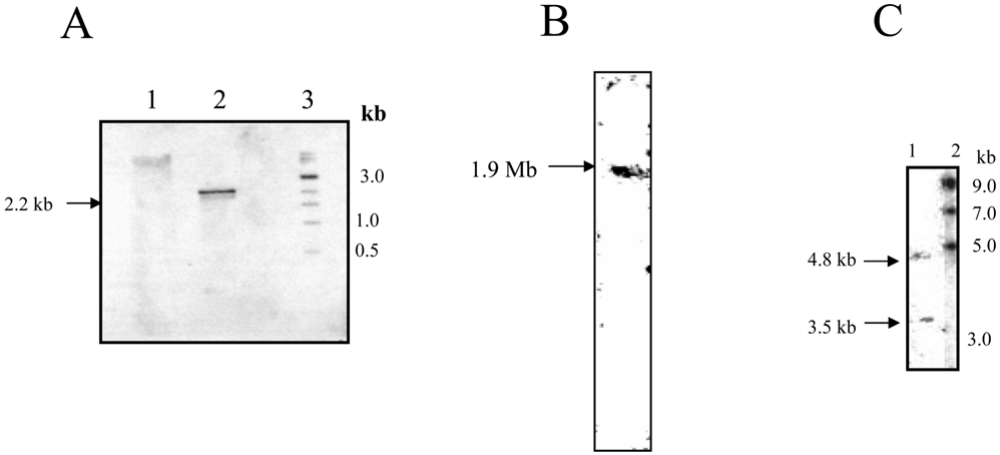
Southern blot analysis of *E. histolytica* putative *ODC*-like gene. (A) Lanes 1 and 2, restriction digest of *E. histolytica* genomic DNA with XhoI and HindIII, respectively. Lane 3 represents the DNA molecular weight marker and sizes are indicated on the right of the figure. The blot was probed with 1,242 bp full-length putative *ODC*-like gene. (B) PFGE analysis of *E. histolytica* indicating chromosomal localization of the ODC gene. The blot was probed with the putative ODC-like gene probe. The arrow shows a 1.9 Mb hybridizing band of putative ODC. (C) Northern blot analysis of mRNA from *E. histolytica.* Lane 1, total RNA from *E. histolytica* probed with 1,242 bp full-length putative *ODC*-like gene; lane 2, molecular weight marker.

To determine the *E. histolytica* putative ODC-like gene copy number, Southern blot studies were performed as described in Materials and Methods, using the 1,242-bp PCR product as a probe (Figure 3). The enzymes used for Southern analysis were Xho I and Hind III, which have no recognition sites in the *E. histolytica* putative ODC-like gene sequence. A single band was obtained in each case (Figure 3A), demonstrating that it is a single-copy gene. A PFGE blot probed with the ^32^P labeled 1,242 bp ODC PCR fragment, hybridized to a 1.9 Mb size chromosome. (Figure 3B). Northern blotting of *E. histolytica* total RNA and PCR-generated ∼1,242-bp gene probe, revealed two transcripts of ∼4.8 and ∼3.5 kb (Figure 3C).

### Overexpression and purification of full-length *E. histolytica* putative ODC-like protein in *E. coli*

**Figure 4:**
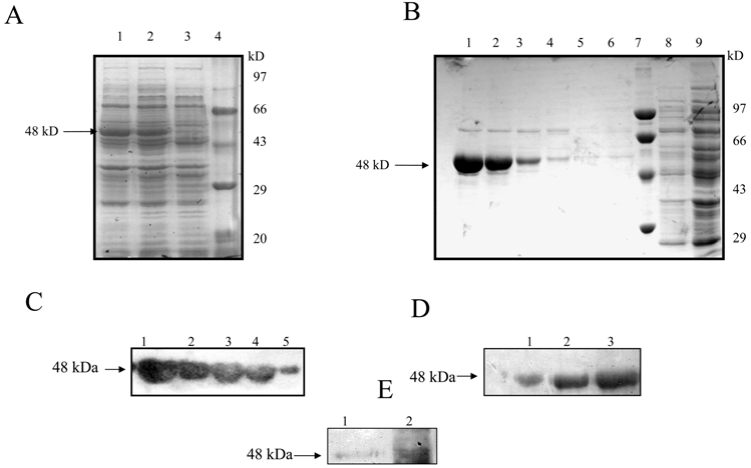
Overexpression and purification of *E. histolytica* putative ornithine decarboxylase-like protein. (A) Coomassie blue staining of SDS/PAGE showing overexpression of full-length *E. histolytica* putative ODC-like protein in *E. coli.* The pET 30a bacterial extract after induction (lanes 1 and 2) at 3 h and 1 h, respectively with 1mM IPTG and before induction (lane 3). The arrow shows the induced recombinant putative ODC-like protein. The broad-range protein MW marker (lane 4) (BioRad) was used to identify the size of recombinant protein. (B) Purification of putative ODC-likeprotein on Ni^2+^ affinity resin. Lanes 1–4, eluted fractions showing purified putative ODC-like protein from affinity column; lanes 5, 6, and 8 are washes; lane 7, broad-range protein MW marker (BioRad); lane 9, supernatant from the crude lysate. (C) Western blot using anti-His antibody. Western blot analysis of different concentrations of purified putative ODC-His fusion recombinant protein. Lanes 1–5 represent 25, 20, 18, 15, and 5 µg of recombinant proteins, respectively. (D) Western blot using anti-*E. histolytica* ODC. Lanes 1–3 represent 2, 3, and 6 µg of purified recombinant protein. Prestained broad-range protein molecular weight marker (BioRad) was used to identify the size of the protein on the Western blot. (E) Western blot using anti-*E. histolytica* ODC. Lane 1, purified recombinant protein. Lane 2, *E. histolytica* lysate. Prestained broad range protein molecular weight marker (BioRad) was used to identify the size of the protein on the Western blot.

In order to characterize the recombinant protein, the gene sequence encoding the *E. histolytica* putative ODC-like protein was cloned in-frame in a pET-30a expression vector with its own start ATG codon. The resultant pET-30a *E. histolytica* putative ODC-like construct was transformed into *E. coli,* and protein expression was induced as described in Materials and Methods. A protein with molecular weight that matched the estimated ∼48 kDa predicted by the amino acid composition of *E. histolytica* putative ODC-like protein with His-tag and S-tag present at its N-terminal end was induced (Figure 4). The recombinant protein was purified on a Ni^2+^-NTA affinity chromatography column (Figure 4A and 4B). To further confirm the size of the protein, a Western blot was done with anti-His antibody that revealed the band of purified product (∼ 48 kDa) (Figure 4C). Recombinant *E. histolytica* putative ODC-like protein was used to raise polyclonal antibody in BALB/c mice as described in Materials and Methods. The antiserum recognized a ∼48 kDa fusion protein on a Western blot of purified recombinant *E. histolytica* putative ODC-like fusion protein (Figure 4D). The same antiserum detected a band of anticipated *E. histolytica* putative ODC at size ∼46 kDa in Western blots of parasite cell extracts, in agreement with the value calculated from the predicted sequence (Figure 4E). Purification of His-tagged *E. histolytica* putative ODC-like protein by metal affinity chromatography yielded ∼3–4 mg of pure protein from a 1-liter bacterial culture.

### *E. histolytica* ODC activity

ODC activity was measured in the crude *E. histolytica* lysates and in the recombinant putative ODC-like protein. Detailed study was limited by its remarkable instability. Addition of dithiothreitol (2 mM DTT), a known stabilizer of mammalian ODC [43], to the purified enzyme samples did not improve the stability of the enzyme or its activity. However, we were able to measure the activity by adding 0.002% BRIJ-35 to the reaction mix. The activity obtained in the crude lysate was 4.8±0.8 nmol h^−1^ mg^−1^ protein, and the recombinant protein gave an activity of 1,311±7.0 nmol h^−1 ^mg^−1^ protein (Table 1). Addition of DFMO (10 mM) to the recombinant ODC protein did not have any affect on the ODC activity (1,085±15 nmol h^−1 ^mg^−1^ protein). The values obtained were not significantly different from that of the control with no DFMO. The K_m_ value for the substrate ornithine was 1.5 mM. The activity obtained here for the recombinant protein was much lower than that reported earlier for the purified protein from *E. histolytica* [20]. Ammonium sulfate purification of the His-tagged recombinant ODC protein from *E. histolytica* did not improve the activity of this recombinant protein. Since we were not able to obtain higher K_m_ values using ornithine as the substrate, we checked substrate preference. Decarboxylation of L-arginine and L-lysine was also measured in order to check the substrate preference of the recombinant protein. In the ODC assay we found no activity using arginine and lysine as the substrate.

**Table 1:** Analysis of ODC activity and polyamine levels of *E. histolytica*

Sample	ODC activity (nmol h^−1^ mg^−1^ protein)	Intracellular polyamine levels (nmol mg^−1^ protein)	
		Putrescine	Spermidine
Crude lysate	4.8±0.8	137±6.4	6.9±0.2

Levels of polyamines in the lysates were detected by high-performance liquid chromatography (HPLC). Results are mean±standard deviation values of three independent determinations.

### Polyamine levels in *E. histolytica*

Analysis of polyamine content of *E. histolytica* revealed substantial levels of putrescine (137±6.4 nmol/mg protein) compared to spermidine, which is present in very low amounts (6.9±0.2 nmol/mg protein). Spermine was not detected in the lysate (Table 1).

### Structural analysis *of E. histolytica* putative ODC

**Figure 5:**
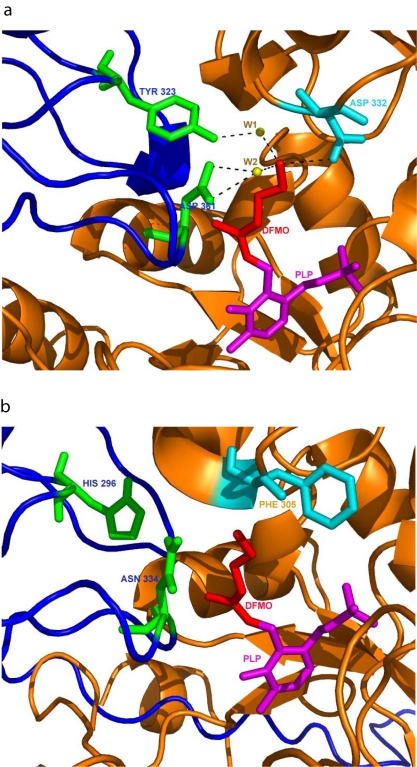
Structural analysis *of E. histolytica* putative ODC. (A) Close up view of DFMO (red) and PLP (magenta) binding sites in the crystal structure of *T. brucei* ODC. The two chains of *T. brucei* ODC are shown in blue and orange. Positions of two water molecules are labeled W1 and W2. Hydrogen bonding is shown in dashed lines. Distances are represented in angstroms (N-ε-of DFMO with W1 : 2.70 Å; W1 with Tyr : 2.75 Å; N-ε-of DFMO with Asp-332 : 2.89 Å; N-ε-of DFMO with W2 : 3.15 Å; W2 with OD2 of Asp 361: 2.99 Å; W2 with OD1 of Asp-361: 2.89 Å) (B) Close-up view of DFMO and PLP binding sites in the modeled structure of *E. histolytica* putative ODC. The color coding is same as mentioned in Figure 5A. The modeled structure was generated using the program MODELLER. The distance between N-ε of DFMO and Asn-334 is more than 3.5Å. Note that the structure with DFMO bound is a deliberate generation of a model corresponding to an unrealistic situation. This figure has been generated using PyMOL [47].

*E. histolytica* putative ODC-like protein is 413 amino acids long, with 32% sequence identity with the ODCs of *T. brucei* and *H. sapiens.* We looked for two important motifs in the sequence of ODC that are essential for binding of DFMO and PLP. The homologues of known three-dimensional structure complexed with DFMO show that the amino acid motif GPSCNGSD corresponds to the binding site for DFMO. DFMO is known to bind to cysteine in this motif. This cysteine is well conserved in the ODC from all three organisms (*T. brucei*, *H. sapiens,* and *E. histolytica*). Despite the presence of the cysteine, *E. histolytica* putative ODC is not inhibited by DFMO. Concentration of DFMO as high as 10 mM did not inhibit the enzyme activity *in vitro.* The lysine in the PCFAVKCN motif is an important residue for the interaction of PLP with ODC, which is well conserved in ODC from all three organisms discussed above. Structural analysis of the *E. histolytica* putative ODC is shown in Figure 5. Known three-dimensional structural analysis suggests that DFMO, which can inhibit *T. brucei* ODC, makes a hydrogen bond (H-bond) with three residues in the two chains of ODC. The nitrogen (ε) of DFMO forms an H-bond with aspartate-332, and water mediates H-bonds with aspartate-361 and tyrosine-323, both from the same chain (Figure 5A). All the three residues mentioned above with which DFMO is interacting are replaced in *E. histolytica* putative ODC. Tyrosine-323, aspartate-332, and aspartate-361 of the *T. brucei* ODC are substituted by histidine-296, phenylalanine-305, and asparagine-334 respectively in the *E. histolytica* putative ODC. The distance between the nitrogen atom (ε) of DFMO and asparagine-334 is more than 3.4Å in *E. histolytica* and hence unable to form an H-bond. These three residues have been labeled in the modeled structure (Figure 5B) of *E. histolytica.*

It should be noted that a deliberately unrealistic model of the *E. histolytica* putative ODC in complex with DFMO was generated in order to understand why DFMO does not bind to *E. histolytica* putative ODC. The overall conformation of modeled *E. histolytica* putative ODC in complex with DFMO and PLP is not very different from that of the *T. brucei* ODC. The site of PLP binding is fully conserved, thus *E. histolytica* putative ODC should be able to accommodate it. It is possible that the substitution of important interacting amino acids in *E. histolytica* putative ODC, makes DFMO unable to bind and hence unable to inhibit the action of *E. histolytica* putative ODC. However, this mechanism can be experimentally proved only by mutating these residues, namely histidine-296, phenylalanine-305, and ssparagine-334, respectively, and determining if the inhibition is restored.

## Discussion

The polyamines putrescine, spermidine, and spermine are polycationic organic compounds present in all eukaryotic cells, including parasitic protozoans. It was reported earlier that the polyamines are essential for the proliferation of normal cells and for differentiation [6].

Ornithine decarboxylase is the rate-limiting enzyme in the de novo synthesis of polyamines and it catalyses the decarboxylation of ornithine to putrescine and is a highly regulated enzyme. Interest in ODC has arisen mainly from the observation that the development of certain tumors closely correlates with increase in enzyme activity and that specific inhibitors of ODC reduce or stop these malignancies [13]. In addition, because of the critical role of ODC in growth and differentiation of the cells, it has been exploited as a target to control certain parasitic infections with specific inhibitors of ODC such as DFMO, a structural analog of ornithine that has been proved as an efficient therapeutic drug [21].

In this paper, we describe the molecular cloning and characterization of putative *ODC*-like gene of *E. histolytica*, a protozoan parasite known for causing amoebiasis. We cloned the putative *ODC*-like gene of *E. histolytica* (GenBank accession number AY929249), and there is only 33% identity to *H. sapiens* ODC. Comparison of the putative ODC-like protein sequence from *E. histolytica* with other eukaryotic species revealed conserved regions. The sequence PFYAVKCN, which resembles the consensus sequences of PXXAVKC(N), contains the lysine (K) to which the pyridoxal 5′ phosphate cofactor binds. Other highly conserved amino acid stretches, for example, FDCAS, EPGR, and FNGF, are also conserved, although their functional significance of these stretches is not known. Phylogenetic tree analysis showed a close evolutionary relationship of ODC of *E. histolytica* and the plant *D. stramonium.* However, comparison of *E. histolytica* putative ODC-like sequences with *L. donovani* and *H. sapiens* showed closer evolutionary relationship with *L. donovani.*

The recombinant putative ODC from *E. histolytica* was very unstable. Addition of 2 mM DTT to the enzyme samples did not improve the activity or stability of this enzyme. Earlier reports also show that purified ODC from trophozoites of *E. histolytica* lost most of the activity after 24 h in unfractionated samples and was reported to be very unstable [20]. In our hands even ammonium sulfate purification of the His-tagged recombinant putative ODC-like protein from *E. histolytica* did not improve the activity of this recombinant protein. The irreversible inhibitor DFMO (α-difluoromethylornithine) did not inhibit activity of *E. histolytica* recombinant ODC (data not shown). Similar observations made previously rules out the possibility of its being used as a suitable target for this enzyme [20].

It has been reported that purified preparations of *E. histolytica* ODC contain a major polypeptide band of 45 kDa and barely detectable amounts of two other proteins of 70 and 120 kDa. Both the 45 and the 70 kDa bands were recognized by a mouse anti-ODC monoclonal antibody [20]. However, in the present study, Western blot analysis of the whole cell lysates of *E. histolytica* using the polyclonal antibody against *E. histolytica* putative ODC-like enzyme showed a single band of approximately 46 kDa, and the same antibody recognized the recombinant protein of about 48 kDa, the expected size of the putative ODC-His tag fusion protein.

Analysis of polyamine content of *E. histolytica* revealed significant levels of putrescine compared to spermidine, which is present in very low amounts. Spermine was not detected in the lysates.

Computer modeling revealed that three of the critical residues required for binding of DFMO to the ODC enzyme are substituted in *E. histolytica* resulting in the likely loss of interactions between the enzyme and DFMO. These residues correspond to Tyrosine-323, Aspartate-332 and Aspartate-361 in *T. brucei* ODC homologue and these are substituted by histidine-296, phenylalanine-305, and asparagine-334 respectively in *E. histolytica* homologue. It is known that Asp-332 and Asp-361 are essential catalytic residues that interact with the substrate [44]. Several members of the ODC family are known to be found in the GenBank database with amino acid substitutions at the position of Asp-332 (D332E) [45]; however, our present study shows that amino acid substitution at Asp-361 (an active site) is unique to *E. histolytica* putative ODC. Asp-332 is highly conserved in the ODC family and is known to play an important role in substrate binding and catalysis. Shah et al. [45] reported that in *Paramecium bursaria* chlorella virus-1 ornithine decarboxylase (PBCV-1 DC) the equivalent position is residue 296, which is glutamate; according to the authors this substitution was a key determinant in the change in the substrate specificity from ornithine to arginine. This substitution (D332E) has also been observed in sequences of antizyme inhibitor, which is an inactive ODC homolog that regulates ODC activity (GenBank accession numbers: human, NP_680479; mouse, NP_06125; and rat, NP_072107) [46]. Furthermore, they investigated the impact of the active-site difference at position 332 on substrate specificity and mutated Asp332 (E296D). They reported that this substitution alone was insufficient to produce the observed substrate specificity change in PBCV-1 DC. In the present study we found a unique *E. histolytica* substitution in the putative ODC-like gene sequence both at Asp-332 and Asp-361; given that the amino acid changes affected the pocket of the *E. histolytica* enzyme, we wanted to know whether it was likely to cause a significant change in substrate specificity. We checked the activity of the recombinant protein using arginine and lysine as the substrate and found no enzyme activity. Kinetic analysis using ornithine as the substrate showed lower kinetic parameters compared to those reported for well-characterized enzymes from other organisms. It is possible that this enzyme has evolved a novel substrate specificity. In view of this situation we feel that this *E. histolytica* protein might have other functions, so far unidentified, including a regulatory role.

In conclusion, characterization of the *E. histolytica* putative ODC-like enzyme and expression of the protein will facilitate studies of structural and functional aspects of the enzyme and could prove to be an important anti-amoebic target.
